# Participants’ perspectives on the medical practitioner compassion competency questionnaire

**DOI:** 10.4102/safp.v67i1.6141

**Published:** 2025-07-15

**Authors:** Willem E. Botha, Michelle Jäckel-Visser, Callie Theron

**Affiliations:** 1 Department of Industrial Psychology, Faculty of Economic and Management Sciences, Stellenbosch University, Stellenbosch, South Africa

**Keywords:** compassion, qualitative research, questionnaire validation, face validity, empathy, mindfulness, compassionate action

## Abstract

**Background:**

The study qualitatively reviewed the Medical Practitioner Compassion Competency Questionnaire (MPCCQ). The revision aimed to extend the questionnaire and address the factor fission found within three subscales of the MPCCQ, namely, mindfulness, emotion recognition, and compassion action orientation.

**Methods:**

A literature review was conducted to inform the development of additional items for the questionnaire. Thereafter, 14 subject matter experts (SMEs) were asked to assess the items in the mindfulness, emotion recognition, and compassion action orientation subscales. Experts provided feedback in an open-ended format, allowing them to freely express any concerns or comments about each item. In addition, they rated each item’s clarity and validity on a scale from 1 (not clear or valid) to 3 (clear and valid). Lawshe’s content validity ratios were calculated to assess the level of consensus among the SMEs and to quantify the need for revision.

**Results:**

Eight items showed statistically significant disapproval from SMEs and were rewritten based on the qualitative feedback from the SMEs. In total, 30 items were amended according to SME suggestions along with previous qualitative data collected by Visser.

**Conclusion:**

The revised questionnaire aims to more accurately and comprehensively capture compassion competency in medical practitioners on the sub-dimensions identified by the original author, ultimately supporting the ongoing development of compassion competency measurement in medical practitioners.

**Contribution:**

In addition, this study contributes to the body of knowledge on qualitative methods for constructing behavioural observation scales.

## Introduction

The 21st century has seen an increase in chronic and lifestyle-related illnesses such as diabetes, obesity, substance abuse complications, hypertension, chronic pain, cancer, and complications from viral infections such as the Human Immunodeficiency Virus (HIV).^1,2^ To manage these lifestyle-related illnesses, medical practitioners have shifted their focus towards illness prevention, wellness promotion, supportive care, and the integration of social and psychological dimensions into the patient profile.^3^ This approach, known as the biopsychosocial model, emphasises person- and patient-centred care.^3^

That said, the construct of empathy, a key component of patient-centred care, has recently been criticised for being an insufficient criterion for evaluating medical practitioners’ patient-centredness. According to Bloom and Ricard, emotional empathy can overwhelm an individual, rendering them unable to assist effectively while cognitive empathy may be used as a tool for manipulation rather than serving as a mediator for effective medical care.^4,5^ To address this shortcoming, researchers have shifted their attention to compassion and argue that the construct incorporates actionable helping behaviour, with a focus on positive emotions associated with caring, thus mitigating the two shortcomings of empathy.^4,5^ Unfortunately, many scales developed to measure medical practitioner compassion still do not differentiate well from empathy and focus narrowly on nurses as the target population.^6^

To address the need for an accurate measure of medical practitioner compassion, Visser developed the Medical Practitioner Compassion Competency Questionnaire (MPCCQ) comprising six structurally inter-related latent dimensions, namely, investing the self, mindfulness, recognition of emotions, gaining and communicating an empathic understanding, caring with kindness, and compassion action orientation.^7^ Ultimately, this tool assesses the competency of medical practitioners to engage compassionately with their patients within the South African healthcare context and can serve as a developmental tool to promote patient-centred care, leading to better compliance and treatment outcomes.^2,8,9^

While the MPCCQ is a promising tool for developmental purposes, it still required revision because the subscales measuring mindfulness, recognition of emotion, and compassion action orientation demonstrated factor fission in Visser’s original study.^7^ Factor fission is the process in factor analysis where a single factor splits into two or more distinct factors because of the presence of multiple underlying dimensions within the data. In the MPCCQ, the factors mindfulness, emotion recognition, and compassion action orientation displayed multifactorial properties and may therefore measure more than one construct. Visser evaluated the factor fission to be theoretically and practically meaningful but did not explore it further.

Consequently, the purpose of this study was to investigate the factor fission identified by Visser by pursuing the following research objectives:

- To establish a theoretical foundation for revising the MPCCQ through a literature review, clarifying the observed factor fission within the mindfulness, emotion recognition, and compassion action orientation subscales.
- To refine the structural model proposed by Visser depicting the internal structure of the medical practitioner compassion construct to ensure alignment with the revised theoretical framework.
- To develop and revise the MPCCQ items in accordance with the updated structural model informed by the literature review that more aggressively taps into the themes unique to the extracted mindfulness, recognition of emotion, and compassion action orientation factors.

The literature review is briefly discussed next, whereafter the revised structural model is described. Finally, the research method for revising the questionnaire is described in detail and the limitations and recommendations for future research are provided at the end of the article.

## Construct clarification and review

### Mindfulness

According to a review by Davidson and Kasznjak, the core components of mindfulness are attention, awareness, memory, and discernment.^10^ However, Voci et al. argue that mindfulness should not be viewed as an analytical awareness but rather as an attentiveness imbued with a kind, non-judgemental attitude towards internal and external experiences.^11^ This form of awareness allows one to fully accept present internal and external experiences without the need to change them.

By evaluating the items present in available open-source mindfulness questionnaires,^12,13,14^ it was found that the items in the MPCCQ mindfulness subfactor reflect both the attention and awareness facet described by Davidson and Kasznjak,^10^ as well as the non-judgemental observing dimension outlined by Voci.^11^ Considering the given information, the definitions of the mindfulness subfactors were slightly revised from the original author’s definition to clarify this distinction^7^:

- *Being psychologically present*: The extent to which the medical practitioner pays attention to the patient, focuses on the encounter and turns off other areas of their lives, resulting in being fully present without distractions.
- *Living in the moment*: The extent to which the medical practitioner is open to the encounter with the patient and accepts and registers what arises in an undistorted, non-judgemental way.

### Emotion recognition

Mier et al. situate emotion recognition within the framework of intention recognition or Theory of Mind (ToM), which is a component of the broader study of social cognition.^15^ This perspective suggests that recognising others’ emotions helps us to understand their intentions. Notably, emotion recognition develops earlier than the ability to understand and interpret emotions, implying that it is an unconscious process.^16^ This is reflected in Coricelli’s ToM model, which consists of unconscious automatic processes – such as emotion recognition, emotional contagion and action recognition – followed by conscious processes such as probing or hypothesis testing, which ultimately lead to a decision about a person’s intentions.^17^

It is theorised that the items in the MPCCQ assess emotion recognition along with both conscious and unconscious processes, as described. The two extracted factors were labelled as recognition and labelling of emotion (factor 1) and emotional probing (factor 2). The definition of the two extracted factors remains unchanged^7^:

- *Factor 1 - Recognition and labelling of emotion*: The extent to which medical practitioners scan the patients’ emotions, accurately interpret verbal and non-verbal cues, and label the emotions without dismissing them.
- *Factor 2 - Emotional probing*: The extent to which medical practitioners probe and explore the emotions that patients experience to gain greater clarity and find the underlying cause.

### Compassion action orientation

In social psychology, compassionate actions are often classified under and used in place of the larger construct called pro-social behaviours. Pro-social behaviour can be defined as a voluntary behaviour intended to benefit another.^18^ This broad construct encapsulates a wide variety of related helping behaviours that Dunfield and Gross et al. categorise into three subtypes according to their function.^19,20^ The three subtypes include helping, sharing, and comforting, which function to provide instrumental needs, provide for unmet material desires, and ease emotional distress, respectively. This model of pro-social behaviours is used to explain the factor fission found in the compassion action orientation subscale of the MPCCQ.^7^

To elaborate, the items loading onto the first subfactor correspond to Dunfield’s comforting subfactor in pro-social behaviours where the aim is to relieve emotional distress.^19^ The other subfactor that emerged corresponds with Dunfield’s helping action intended to aid instrumental needs and was labelled as organising resources.^19^ Formally, the two subfactors are defined as follows^7^:

- *Organising resources*: The extent to which medical practitioners assist patients with realising their wishes, solving their problems, and making use of other resources in an attempt to aid in solving their needs.
- *Compassion action*: The extent to which medical practitioners assist patients in coping with the distress and suffering associated with their illness and in making sense of what is happening, with the intent to provide some comfort to the patients.

### Refining the Medical Practitioner Compassion Competency Questionnaire measurement model

**FIGURE 1:**
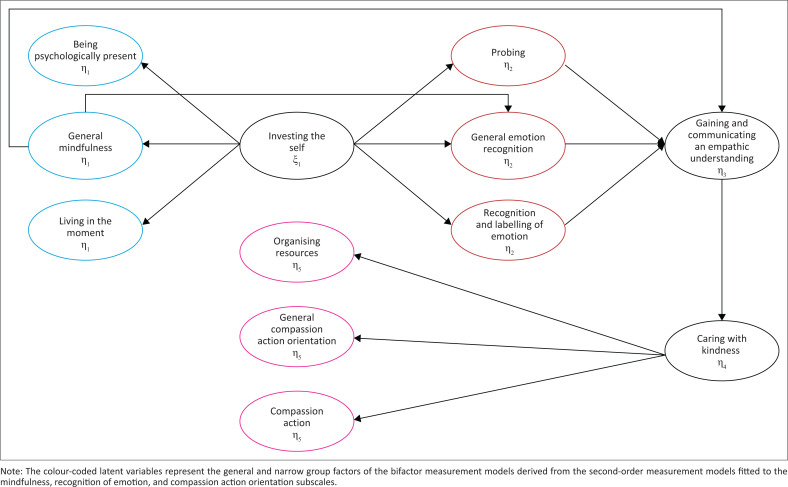
Internal structure of the refined compassion competency construct based on the Schmid-Leiman subfactors.

The literature review clarified the connotative meanings of the subfactors mindfulness, emotion recognition, and compassion action orientation, providing a theoretical basis for refining the MPCCQ measurement model. However, the extent to which these subfactors function as distinct constructs remained unclear and were subsequently examined by applying the Schmid-Leiman transformation to the completely standardised second-order factor measurement model parameter estimate data obtained by Visser.^21^ Results indicated that a general factor representing the variance shared by the extracted first-order factors accounted for over 82% of the explained variance in item responses for all three subscales. This suggests that, while the connotative meaning of the subfactors may be theoretically and practically important, the explained subscale item variance was largely because of a general factor rather than the residualised first-order factors representing the themes unique to the extracted first-order factors. Accordingly, it was deemed more appropriate to refine the structural model using a bifactor structure, wherein a general factor accounts for the majority of explained item variance, alongside two uncorrelated residualised factors that manifest only in specific items. The refined structural model is presented in Figure 1.

The new structural model includes additional factors, and the original questionnaire developed by Visser does not have enough items to accurately measure the latent constructs that emerged from the factor fission as conceptualised in Figure 1. Consequently, the study followed Visser’s recommendation to develop three to four additional test items per subfactor to ensure there are at least five items per subscale. The additional items were specifically developed to tap into the themes unique to the extracted first-order factors rather than the (general) theme shared by the first-order factors. Given the theoretical background, and the information available from the original study by Visser, the researchers decided to employ both inductive and deductive approaches in item development. The literature review guided the constructs each item should address (deductive item development), while transcripts from interviews conducted by Visser provided the specific contextual content for each item (inductive item development).

As per best practice guidelines, the refined questionnaire should be reviewed by a small group of subject matter experts (SMEs) to assess the relevance of the items and the overall suitability of the questionnaire.^22,23^ The following section details the research methodology used to evaluate the refined MPCCQ.

## Research design and method

### Research design

The objective of the qualitative review is to revise the MPCCQ items in accordance with the updated structural model informed by the literature review. This is achieved by:

- Reviewing the face validity of the items.
- Reviewing the clarity of the items within the revised MPCCQ.
- Rewriting items that may be unclear.
- Rewriting items that predominantly reflect the general factor and not the subfactors described in the literature review.

The most appropriate way to achieve the above objectives is through expert reviews by SMEs. Given that the data involves expert opinion, a qualitative research method is the most appropriate. Qualitative methods are used to gather rich, detailed data that allow for a flexible and thorough exploration of each participant’s perspectives. To enhance the rigour of the research, participants were also asked to rate the items following the approach used by Lawshe and Nel.^24,25^ Integrating a quantitative approach for data triangulation aligns with the post-positivist paradigm of measuring objective reality, ensuring that decisions regarding item revisions are not solely based on the subjective interpretation of qualitative data, but are supported by accurately quantifying participants’ opinions.

### Research method

Subject matter experts were asked to rate each item on the revised subscales of the mindfulness, emotion recognition, and compassion action orientation based on their clarity and validity on a scale from 1 to 3, as shown in Table 1.

**TABLE 1:** Qualitative evaluation criteria.

Definition	Rating	Rating description
Validity: The permissibility of inferences derived about a medical practitioner’s standing on a latent compassion competency from the behaviour described in the item.	1	It is not permissible to infer a medical practitioner’s standing on this latent compassion competency from their response to this item.
	2	It is partially permissible to infer a medical practitioner’s standing on this latent compassion competency from their response to this item.
	3	It is definitely permissible to infer a medical practitioner’s standing on this latent compassion competency from their response to this item.
Clarity: The item can be understood easily, i.e. the syntax and semantics are appropriate.	1	The item is unclear.
	2	The wording of the item requires several modifications or a very large modification in terms of meaning or word order.
	3	The item is clear, with appropriate semantics and syntax. Very little modification is needed, if any.

*Source:* Adapted from: Fernández-Gómez E, Martín-Salvador A, Luque-Vara T, Sánchez-Ojeda MA, Navarro-Prado S, Enrique-Mirón C. Content validation through expert judgement of an instrument on the nutritional knowledge, beliefs, and habits of pregnant women. Nutrients. 2020;12:1136

If the participants rated any item below three for clarity or validity, they were requested to provide open-ended comments about the items, whereafter the researcher altered the item content based on these suggestions depending on the level of agreement between SMEs. The level of agreement between SMEs was evaluated using an aggregate score of their ratings, called the content validity ratio (CVR) of an item, which ranges from 1 to +1.^25^

A value of 1 indicates that all SMEs found the item clear and/or valid; a value of 0 means half of the SMEs agreed on its clarity and/or validity; and a value of –1 suggests that all SMEs found the item unclear and/or invalid. In this study, a CVR value below 0.54 indicates statistically significant disapproval (one-tailed, *p* < 0.05) by SMEs with regard to the appropriateness of the item, suggesting that alterations to the item are necessary.^25^ The item was rewritten if either the clarity or validity CVR reflected problems with the item. It is important to note that the goal was not to achieve complete agreement among SMEs, but rather to revise items that received unanimous disapproval from them, as evaluated by the CVR.

**FIGURE 2:**
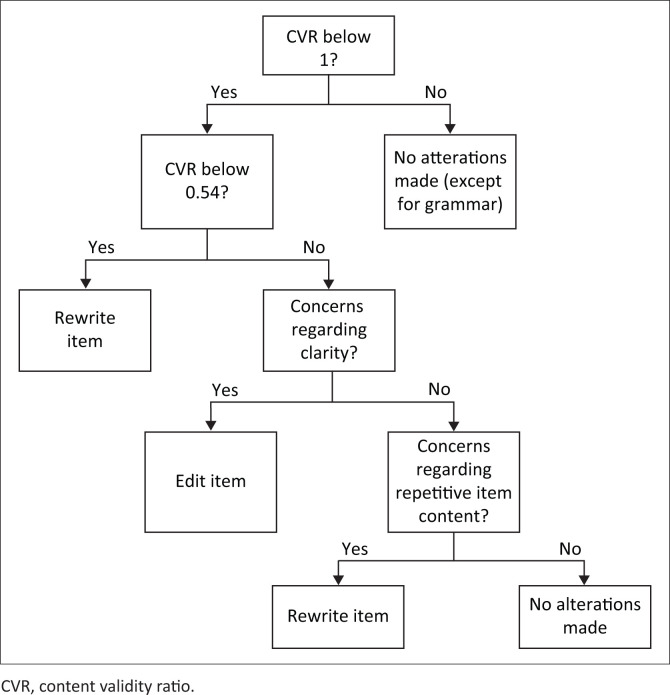
Decision tree used to evaluate the extent of alterations required for each item.

However, slight modifications were made to items to eliminate any potential confusion about what was being asked, even if the CVR was above the cut-off value. However, in those cases, care was taken to ensure that these modifications did not alter the content of the items. Furthermore, if the item was deemed to be repetitive by any participant, then the item was rewritten. Figure 2 summarises the item alteration process.

The SMEs were not asked to re-evaluate the new and revised items because of time constraints and concerns about overburdening participants. Ideally, item revision should continue until a consensus is reached among SMEs. Nevertheless, the researchers carefully implemented the SMEs’ feedback, taking care to avoid introducing their own biases into the items.

### Sampling strategy

Purposive and snowball sampling were deemed the most appropriate methods for participant selection. After the selected SMEs were approached to participate in the study, they were asked to refer other relevant individuals in their field to participate. The researchers aimed to recruit a minimum of five SMEs to participate in the study as this allows for the implementation of Lawshe’s CVRs.^25^ However, a larger sample size enhances the confidence with which the results can be considered genuinely reflective of expert opinions; consequently, the researchers encouraged and allowed a greater number of participants to participate in the study. By the end of the data-gathering phase, 14 SMEs had completed the review, and this was deemed sufficient.

### Inclusion criteria

While the target population for the MPCCQ is medical practitioners working in the public healthcare sector in South Africa, including experts in fields related to questionnaire development such as psychometry, industrial psychology, and language editing may increase the chances of identifying problematic items that may cause confusion because of possible ambiguous or compounded item presentation. This research, therefore, had the following inclusion criteria:

- Industrial psychologists, research psychologists, or psychometrists registered with the Health Professions Council of South Africa (HPCSA). The aforementioned professionals ideally had to have previous experience in training or working with individuals employed within the healthcare sector.
- Linguists or experienced language professionals with a background in proofreading and/or editing. The language professionals ideally had to have completed courses in psychology and/or psychometrics.
- Licensed medical practitioners under the HPCSA, actively practising in South Africa. In addition, they should have completed the requirements of their designated internship and community service years and have specialised in one of the following core disciplines: family medicine, internal medicine, paediatrics, obstetrics and gynaecology, and surgery.^7^

### Sample composition

Table 2 provides a detailed description of the sample characteristics and shows that the participants varied greatly in terms of their years of experience, ranging from three years to 40 years, with half of the participants (*n* = 8) having between three years and 10 years of experience. Furthermore, nine of the 14 participants’ home language was Afrikaans, four participants’ home language was English, and one participant did not specify. The majority of the sample (seven participants) were from family medicine, two participants were from industrial psychology, and there was one participant each from pathology, psychiatry, psychometry, language editing, and obstetrics and gynaecology. Two participants were with specialisations in the fields of psychiatry and pathology, therefore, they fell outside the inclusion criteria. Despite this, they were included in the final data analysis because the pathologist had extensive experience working in South Africa’s public healthcare sector and the psychiatrist had substantial knowledge within the field of psychology. Thus, the final sample comprised 71% medical practitioners and 29% specialists in test development.

**TABLE 2:** Summary of sample composition with additional noteworthy characteristics.

P	YoE	HL	Speciality	Additional important characteristics
1	3	English	Psychometry	Assists with research related to healthcare and psychometric test validation.
2	9	English	Language editing	Is currently completing their master’s programme in psychology.
3	4	Afrikaans	Industrial psychology	Experienced in soft skills training for medical students.
4	36	Afrikaans	Obstetrics and gynaecology	-
5	22	Afrikaans	Family medicine	Researcher and trainer in medical practitioner compassion.
6	26	Afrikaans	Family medicine	-
7	32	Afrikaans	Family medicine	-
8	13	Afrikaans	Family medicine	-
9	-	English	Family medicine	-
10	6	Afrikaans	Psychiatry	-
11	6	Afrikaans	Industrial psychology	Specialises in medicolegal reports.
12	33	English	Family medicine	Does training with medical practitioners on ‘breaking bad news’ to patients.
13	10	Afrikaans	Pathology	Academic in the public healthcare sector.
14	-	-	Family medicine	-

P, participant; YoE, years of experience; HL, home language; ‘-’, indicates that there is no relevant information available, or that the information was omitted.

## Ensuring trustworthiness

This study sought to ensure trustworthiness in the qualitative research process by addressing the criteria of credibility, confirmability, transferability, and dependability.

### Credibility

In this study, the researcher provided the full response of each participant in Online Appendix 1. The participants’ views are therefore documented accurately and transparently. In addition, the Lawshe rating method was employed alongside the qualitative feedback as a triangulation method whereby the participants’ views were both recorded and quantified.

### Confirmability

In the context of this study, confirmability is achieved when the researcher can demonstrate that the alterations made to the item content are substantively derived from the qualitative data. This study took the following steps to ensure confirmability:

- Figure 2 details the decision tree used for altering item content.
- The reader can consult Online Appendix 1 to review the actions that were taken for each item based on the qualitative feedback provided by each participant.
- When an alteration was not based on the qualitative feedback provided by the participants, quotations from the critical incident technique (CIT) conducted by Visser were used and were indicated as such.
- All alterations made to each item are documented in Online Appendix 1, and compared to the original item in Online Appendix 2.

### Transferability

It is generally difficult in qualitative research to demonstrate that findings can be applied to other contexts, and as such, it is often the case that the researcher specifies the cases to which the results can be applied. This is carried out by documenting factors such as sample composition, the number of participants involved in the research, the data collection methods employed, and the time period over which the data were collected.^26^ These characteristics are observed in this study.

### Dependability

To ensure dependability, every step of the analysis was thoroughly documented, and the methodology was explained in detail. A table meticulously documenting each alteration to the items can be consulted in Online Appendix 1. Consequently, future researchers should be able to replicate the study documented here and assess whether this study adhered to proper research practices and protocols.

### Ethical considerations

An application for full ethical approval was made to the Social, Behavioral and Education Research Ethics Committee (REC: SBE), Stellenbosch University and ethics consent was received on 15 February 2024. The ethics approval number is 27193.

To protect the participants’ anonymity, they were not required to record identifiers on their questionnaires, which were instead assigned numbers that could not be traced back to them and sensitive demographic information unrelated to the evaluation of the questionnaire’s content was not requested. However, to ensure scientific rigour and allow for the replication of the study by other researchers, the following demographic information that might influence the SMEs’ ratings of the scale was collected:

- core discipline
- years of experience
- home language.

Written consent was obtained by means of an informed consent form. This form explained the study’s aim, nature, procedure, benefits, confidentiality, and complaint procedures. The informed consent form was emailed to the SMEs along with the questionnaire. Stellenbosch’s Social, Behavioural and Education Research Council approved the study on 15 February 2024 (Project number 27193).

## Results

Online Appendix 1 demonstrates how the results were processed. Columns 2 and 3 present the degree of agreement between SMEs as evaluated by the CVR. Any value below 0.54 is considered a statistically meaningful disapproval of item content.^25^ Overall, eight items had CVR values below 0.54 and were rewritten. A total of 30 items were amended. The individual concerns generally fell under the following broad categories:

- Wording ambiguity: The words used in the items lend themselves to more than one interpretation, and the participant has to exert mental effort to contextualise the phrase.
- Grammatical errors: Spelling errors, grammatical errors, and punctuation.
- Sentence structure: The sentence structure is verbose and needs simplification.
- Inappropriate content: The content of the item may not be an appropriate indicator of the construct being measured.
- Overlapping content: The content of the item is not clearly distinguishable from other items. The content of the item is repetitive.

Although the issues primarily involved syntactic and phrasing ambiguities, the significance of these factors in psychometric research should not be overlooked. Previous research has shown that simply changing a statement into its negative form (i.e. by adding the word ‘not’) can severely impact the factor structure and inferences made from the measuring tool.^27^

To assess the potential impact of clarity and validity on the items’ functioning, one can compare the average CVR of the original items with the reliability estimates calculated by Visser. This comparison is presented in Table 3, where a clear relationship between the combined average item CVR (column 2) for the original items and the reliability estimates (columns 3 and 4) is evident. Mindfulness, which had the lowest combined CVR score (0.71), also showed the lowest reliability estimates, with a Cronbach’s alpha of 0.736 and a Multidimensional Omega of 0.603. In contrast, emotion recognition had the highest combined average item CVR (0.86) and correspondingly the highest reliability estimates, with values of 0.832 and 0.792. Finally, compassion action orientation’s combined average item CVR (0.79) fell between those of the factors mindfulness and emotion recognition, and its reliability estimates similarly fell between those of the other two factors, with a Cronbach’s alpha of 0.803 and a multidimensional omega of 0.715.

**TABLE 3:** Comparison of subscale content validity ratios with traditional factor reliability estimates.

Original scale items	Combined average Item CVR	Cronbach’s α	Multidimensional ω
Mindfulness	0.72	0.736	0.603
Emotion recognition	0.88	0.832	0.792
Compassion action orientation	0.84	0.803	0.715

CVR, content validity ratio.

While these relationships are promising indicators that the items’ clarity and validity may have influenced the reliability estimates of these scales, they do not constitute a statistical confirmation thereof. The revised questionnaire needs to be redistributed, and new reliability estimates need to be evaluated for a more definitive evaluation of the impact that the alterations may have on the overall reliability of the scale.

## Discussion

The qualitative data were not subjected to formal thematic analysis, as the open-ended feedback lacked the depth required for such an approach. Instead, the feedback was used directly to inform revisions to the item content during the review process. Nonetheless, consistent and conflicting concerns raised by various SMEs are outlined below, highlighting common pitfalls in item development that future researchers should consider.

### Utility of diverse subject matter experts sample

When analysing the qualitative feedback from participants, it became clear that their educational backgrounds influenced the content of their feedback. The psychometrist and language editor were particularly thorough in scrutinising sentence structure, readability, and the function of the wording. For instance, the psychometrist made an insightful observation about the value of the phrase ‘as time does not allow for this’ in item E1 aimed at measuring emotion recognition:

‘For statement 1, I would remove “as time does not allow for this.” This seems irrelevant to what you are trying to measure, and it may not register as true for all participants, regardless of their emotional attentiveness.’ (P1)

Indeed, the phrase, ‘as time does not allow for this’ may be more appropriate for the factor compassionate action, because it is often the case that medical practitioners view short consultation times as an unavoidable consequence when they are required to attend to numerous patients per day, and this is not necessarily an indication of emotion recognition.^9^ Similarly, the language editor’s detailed understanding of the English language is evident in her feedback on item M12:

‘In option 1, correct to “When seeing a patient, I very seldomly …” Also, add a full stop at the end of each item. In options 3 and 5, add a comma after “When seeing a patient.” I think the word “comprehension” should be replaced with “understanding” in all three options.’ (P2)

In contrast to the feedback from the language and psychometry specialists, the medical professionals were less focused on the precise wording of the items and more concerned with their applicability to their specific context. Based on their personal experiences, they found that some items might make unreasonable demands on medical practitioners. For example, consider the feedback from the medical professionals P4, P5, and P6 on item M7 regarding note-taking being perceived as a distraction:

‘Is taking notes during a consultation regarded as distracting? I make notes and it helps me to focus.’ (P4)

‘Just wondering if there is a different way of describing “to write a lot of notes” in option 1. The issue is not the extent of the note-taking and documentation but how distracted the doctor is or how note-taking detracts from the doctor’s ability to be attentive to the patient. Even writing sparse notes could also be done in a distracted manner – it is not the length of the notes but the process of writing notes which may impact the clinician’s ability to be attentive. Many doctors do not write extensive notes but stick to the bare minimum for medicolegal purposes.’ (P5)

‘Notes vary according to patient and diagnosis. It can be a lot or a little. I am hesitant to quantify the amount of notes.’ (P6)

As an additional example, note how medical practitioners highlighted potential confusion with the phrase ‘resistance to treatment’. While industrial psychologists and psychometrists naturally interpreted the phrase as referring to behavioural or emotional resistance, the doctors pointed out that it can be understood as biological resistance:

‘This may need better explanation as some medical practitioners might interpret resistance to treatment as biological resistance or non-response to treatment. Maybe wording it as resistance to “engaging” in treatment might make it clearer.’ (P10)

‘Would change domain to probing treatment resistance.’ (P13)

The rich qualitative data collected by the research participants indicate that it was a fruitful exercise to include SMEs from various educational backgrounds to review the questionnaire. As a whole, they were able to provide feedback on both technical and practical issues with the items.

### Consequences of narrowly defined factors

When more narrowly defined constructs are used, important information relating to the general construct may be overlooked. Conversely, when more general items are used, it makes it more difficult to provide differential feedback on specific competencies that require improvement.^27^ Participant P5 was particularly perceptive to this and raised concerns that focusing on emotion recognition may exclude important considerations that influence patient care. Take the following comments as an example:

‘In motivational interviewing or brief behaviour change counselling, we consider many factors [*other than emotions*] which affect decisions related to engaging with health behaviour change decisions. I wonder if the word “emotions” may be deemed too unidimensional/limited. We could rephrase “emotions” to ideas, fears, or concerns?’ (P5)

‘See comments above regarding additional considerations which affect behaviour and treatment adherence.’ (P5)

While this is valuable input, generalising the scale to include broader aspects of intention recognition as opposed to emotion recognition may ultimately result in the multidimensional construct collapsing into a general construct. In other words, differential feedback on emotion recognition specifically will not be possible if the item is more generalised. The items were therefore left unchanged.

That said, an additional, and very important point highlighted by participant P5, is that the narrowly defined items may be too similar. Because the facet being measured is so narrowly defined, creating items with a clear distinction from one another can be difficult. For participant P5, this was particularly true for the facet of attention and awareness. Similarly, participant P11 found that the items probing emotion recognition competencies may be too similar and fail in their task to extract unique information:

‘I am unclear about how M9 differs from M7 and M8. Perhaps it would be useful to define the differences more clearly?’ (P5)

[With regard to item E4] ‘Repetitive, as previous items’ answers should provide the same information.’ (P11)

Given the danger of asking the same question more than once, items M7, M8, E1, and E4 were rewritten to better differentiate themselves from other items. The final aspect highlighted by the SMEs was their overall opinion of the questionnaire. Their feedback primarily focused on two themes: the length of the questionnaire and its overall utility. These themes are discussed in the following sections.

### Questionnaire length and administration

It is important to acknowledge the time it took respondents to complete the questionnaire, as this can have an impact on its practical administration. Specifically, the questionnaire’s length may affect the response completion rate, the viability of re-assessments, the user experience, and the productivity of employees who need to take time off work to complete it (when used in an HR context). Three of the SMEs expressed concerns about the questionnaire’s length, as highlighted by the following quotes:

‘You will see in the comments that it took me a bit longer than 30 minutes to work through this – firstly, because the first few questions required a bit more effort from my Afrikaans brain to understand, and secondly, because I also reflected a bit on my own compassion competence.’ (*English translation*) (P4)

‘It took me much longer to complete the task because on almost all the questions I reflected on my own practice and what I do.’ (P4)

‘I think the phrasing is just a bit long-winded at times.’ (P1)

‘Very tiring questionnaire.’ (P7)

The responses indicate that the questionnaire may take longer to complete for individuals whose home language is not English. In addition, although the items need to be very specific, this specificity can make them cumbersome to read and sometimes feel long-winded to the participants. This feedback is particularly concerning because participants were only asked to evaluate 9 of the 12 factors. Thus, it may take participants up to an hour or more to complete the full questionnaire, raising concerns about its practicality and frequency of use among healthcare professionals who are often overworked and under pressure.

### Utility for self-development

Despite concerns about the questionnaire’s length and verbosity potentially affecting its administration and completion rate, respondents still found the content insightful. In fact, some respondents noticed that completing the questionnaire was personally enriching, allowing them to critically reflect on their behaviours in ways they had not performed before. Take the below quotations as an example:

‘Thorough questionnaire. Seems like it will be a helpful measuring tool.’ (P14)

‘Very thorough and thought provoking – it will serve the purpose well.’ (P9)

‘Generally very good – thorough & clear.’ (P12)

‘Very insightful […] I almost think practitioners should do this questionnaire at regular intervals to assess compassion competency.’ (P4)

From the feedback provided, it can be concluded that the questionnaire potentially achieves its purpose of promoting self-reflection.

### Limitations

The sample comprised participants mostly from family medicine, and most of them were native speakers of Afrikaans or English. Consequently, the perspectives of practitioners in paediatrics, internal medicine, and surgery – as well as those whose home language is not Afrikaans or English – were not included in this study. This study did not administer the questionnaire and the reliability and validity of the new measurement model were therefore not tested.

### Recommendations

It is recommended that the revised questionnaire be administered to medical practitioners in South Africa in order to properly validate the questionnaire. To validate the structural and measurement model of the MPCCQ, the researcher should use well-established techniques such as exploratory and confirmatory factor analysis, along with structural equation modelling. It is acknowledged that in the validation of the revised MPCCQ, the operationalisation of the six narrow factors related to mindfulness, recognition of emotions, and compassion action orientation will present an interesting methodological challenge. Moreover, obtaining scores on these narrow (residualised) mindfulness, recognition of emotions, and compassion action orientation factors to unlock the practical value of formative feedback for medical practitioners on the themes unique to the extracted first-order factors will present an even more exciting methodological challenge.

## Conclusions

Based on the literature review and qualitative research, the study successfully achieved its objectives of establishing a theoretical foundation for revising the MPCCQ and refining the constitutive definition of compassion for medical practitioners through adjustments to the construct’s internal structure. In addition, the study reviewed both new and existing MPCCQ items with SMEs, resulting in the revision of 8 items deemed unsatisfactory by the majority of experts, and minor amendments to 30 items. The study ultimately resulted in a revised MPCCQ with more clearly written items, which should, theoretically, assess more refined constructs.
